# Seeing is believing: new methods for *in situ* single-cell transcriptomics

**DOI:** 10.1186/gb4169

**Published:** 2014-03-31

**Authors:** Gal Avital, Tamar Hashimshony, Itai Yanai

**Affiliations:** 1 Department of Biology, Technion – Israel Institute of Technology, Haifa 32000, Israel

## Abstract

New methods employ RNA-seq to study single cells within complex tissues by *in situ* sequencing or mRNA capture from single photoactivated cells.

## Introduction

An old adage relates how a drunk looks for his car keys under a lamppost in the middle of the night - when asked why he searches for the keys many meters away from his car, where they presumably lie, the drunk answers that it is ‘too dark over there’! The transcriptome - defined as the set of all transcripts in a given sample - can be cartooned as that lamppost. Unlike the proteome or the metabolome, the transcriptome has the unique property of being quantifiable thanks to the relative ease of working with nucleic acids. Microarrays first took advantage of strand complementarity in the 1990s for global gene-expression measurements, and, more recently, with drops in the cost of sequencing, RNA sequencing (RNA-seq) is now commonly used to profile the transcriptome with unmatched resolution. Two new single-cell transcriptomics methods further advance RNA-seq by empowering the study of transcripts in their native environments [1,2].

## The complexities of transcriptomics

As bright as the light cast by the transcriptome appears, it must be acknowledged that it does not always translate to biological function. Nonetheless, the transcriptome is attractive because it is arguably the first phenotype of the genome and it comprises the many recently recognized groups of RNA, such as long non-coding RNAs and microRNAs, in addition to protein-coding RNAs.

Examining transcriptomes in a complex, multicellular sample often involves painful compromises. For example, when analyzing the expression profile of a gene in an embryonic time-course, not knowing which specific cell types within the embryo are expressing the gene is a severe handicap. Harnessing methods that can monitor gene expression at the level of individual cells thus holds great promise for resolving the details of cell-type-specific expression. Moreover, even in a population of a given cell type, variation in gene expression can illuminate important functional characteristics. For example, Shalek *et al.* recently examined 18 cells and found extensive evidence for bimodal gene expression within a population of immune cells [3]. Characterizing such heterogeneity at a global level could further expose unsuspected regulatory mechanisms.

**Figure 1:**
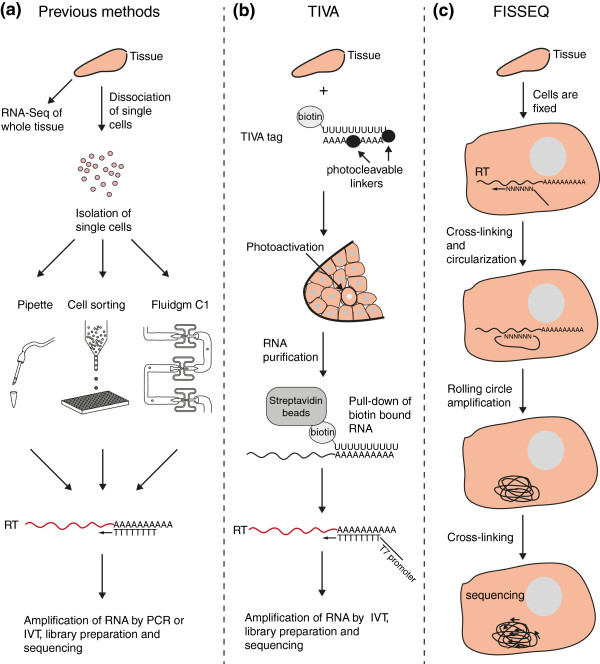
**New methods enable single-cell transcriptomics profiling of a complex tissue. (a)** In single cell approaches, canonically, individual cells are first dissociated and then isolated by micropipette, automated cell sorting, or microfluidics-enabled capturing. Cellular RNAs are then amplified and sequenced. **(b)** The transcriptome of an individual cell can be determined by first photoactivating a TIVA-tag within that cell, allowing it to hybridize to cellular mRNAs. These mRNAs can then be specifically purified and sequenced by any RNA-seq method. **(c)** FISSEQ allows for RNA-Seq within the context of the cell by first fixing mRNA and then amplifying (by rolling circle amplification) and sequencing by the SOLiD technology. FISSEQ: fluorescent *in situ* RNA sequencing; IVT: *in vitro* transcription; PCR, polymerase chain reaction; RNA-seq: RNA sequencing; RT: reverse transcription; SOLiD; sequencing by oligonucleotide ligation and detection; TIVA: transcriptome *in vivo* analysis.

An amplification step exploiting the strand complementarity of nucleic acids is necessary in all single-cell transcriptome methods (Figure 1a-c), including those discussed below. In this context, James Eberwine first introduced transcriptome amplification using T7 *in vitro* transcription (IVT) [4]. For a single cell, three rounds of IVT are required, yet the advantage of this approach remains, in that amplification is linear, as opposed to the exponential phase of PCR. IVT was used widely in most gene-expression microarray platforms. With the introduction of RNA-seq, samples can be molecularly barcoded, as in a recently introduced CEL-Seq method, allowing for many single cells to be processed in parallel and thereby requiring only a single round of IVT [5]. PCR-based single-cell transcriptome methods are also available, each with distinct advantages [6-8]. More recently, the notion of unique molecular identifiers (known as UMIs) was introduced to tag transcripts before the reverse-transcription step, allowing for measurement of individual transcripts with dramatically reduced amplification bias [9].

## Single-cell transcriptomics in a complex tissue

In many projects, a particular cell type in a population constitutes a rare subset. Examining these cells individually might mean excising them from their local tissue environment and thus compromising the quality of their transcriptomes. A recently published method from Lovatt *et al.* now allows examination of the transcriptome of an individual cell in a complex tissue by simply shining a laser upon it [1]. This is made possible by washing a live tissue with a transcriptome *in vivo* analysis (TIVA) tag, a cleverly designed molecule that, like a Swiss Army knife, possesses many properties: a cell-penetrating peptide, a photocleavable linker, the fluorophores Cy3 and Cy5, a poly(U) oligonucleotide and biotin (Figure 1b). Thanks to its cell-penetrating peptide, the TIVA tag can permeate into the cells of a tissue; however, by design, this entry causes the peptide to dissociate from the TIVA tag, trapping it in the cell. Although all the cells in the tissue will contain TIVA tags, these tags will not hybridize to cellular mRNAs as their poly(U) oligonucleotides, which bind to mRNA poly(A) tails, are normally hidden. Nevertheless, laser photoactivation on a particular cell causes its TIVA tags to come undone, as can be validated by fluorescence resonance energy transfer through exploiting the Cy3 and Cy5 molecules on the tag. Once exposed, these tags can then anneal to mRNAs in the light-selected cell and, after harvesting RNA from the entire tissue, the desired mRNA can be pulled out by using streptavidin beads and then be sequenced (Figure 1b).

Lovatt *et al.* applied TIVA to study gene expression in brain slices from mouse and human, where they detected expression consistent with neuronal cell types [1]. Most interestingly, significantly more bimodal expression was recorded in cells isolated from live tissues in comparison with single neurons in culture, providing additional examples of the bimodality first characterized by Shalek *et al.*[3]. An especially useful aspect of the TIVA method is that it can be used to connect the particular morphology of a cell to its transcriptome. The TIVA method is thus the first approach to allow for the study of the transcriptome in cells within intact tissues. This provides a significant advance over methods such as laser capture or isolation by pipette (Figure 1a), which can either injure the cell in the collection process or only partially collect the cell - relevant especially for cells, such as neurons, that possess complex morphologies.

## *In situ* sequencing for all transcripts in individual cells

As mentioned above, single-cell transcriptomics requires amplification, a step that, until now, has involved RNA collection and thus loss of the cellular context of each RNA. In cases where cellular localization information is desired (of mRNA or protein), *in situ* hybridization is an option, but the capacity of this approach is limited to only a handful of genes at a time. Recently, Ke *et al.* introduced a method for sequencing RNA *in situ* and showed that short 4-bp reads can detect point mutations in selected genes [10]. Meanwhile, Lee *et al.* have now demonstrated a novel approach that takes this a dramatic step forward by profiling the transcriptome *in situ* in fixed cells, in a method termed fluorescent *in situ* RNA sequencing (FISSEQ) [2]. This science-fiction-like method relies on performing RNA-seq directly in the cells of interest, through a combination of *in situ* RNA amplification in a fixed sample and sequencing by oligonucleotide ligation and detection (SOLiD).

FISSEQ involves the reverse transcription of RNAs in fixed cells with dNTPs enriched with aminoallyl-modified dUTPs (aaUTPs; see Figure 1c). These cDNAs are then fixed to the cell protein matrix using a crosslinker that exploits the NH group of the aaUTPs. Rolling circle amplification on the fixed cDNA produces a long single-stranded cDNA for each transcript that can then be sequenced *in situ* using the SOLiD sequencing platform.

Lee *et al.* show that the amplification step is able to work in mouse embryos, mouse brain, *Drosophila* embryos, HeLa cells, human primary fibroblasts and human induced pluripotent stem cells. For fibroblast cells, the authors also sequence amplicons and report a transcriptome visualized within the confines of cellular membranes. The SOLiD sequencing of these cells yields 27-bp reads for 15,000 amplicons covering 4,000 genes. As the reverse-transcription step begins with random hexamers (as opposed to the poly(U) oligonucleotides often used in other protocols, including TIVA), the transcriptome is expected to include a sizeable fraction of non-mRNA transcripts. Yet, for fibroblasts, Lee *et al.* reported that 44% of transcripts were mRNAs, which could be attributed to the enrichment of distinctly localized mRNAs that are more resolvable by FISSEQ. When the transcriptome was again probed during simulated wound healing, the mRNA fraction dropped to 7%. Comparing these transcriptomes, the authors found that the differentially expressed genes matched those previously implicated in wound healing.

A comparison of FISSEQ with traditional RNA-seq showed that the two methods were generally well correlated. However, the correlation was notably poor for RNAs involved in RNA and protein processing, leading the authors to make the interesting suggestion that these RNAs localize to cellular structures that are effectively inaccessible to FISSEQ. The ability to establish the localization of gene expression in many cells in parallel will likely make FISSEQ the method of choice for many single-cell transcriptomics applications.

## Discussion

The holy grail of single-cell transcriptomics is to identify the transcript abundance of all genes in a cell positioned in its native environment across both space and time, and to do so without compromising the viability of the cell. Along these lines, TIVA constitutes a major step forward and should prove invaluable in identifying intercellular relationships by enabling analyses of the transcriptomes of neighboring cells at a high level of resolution. Also among the strengths of TIVA as a technology is its accessibility as it appears reasonably friendly to the uninitiated, relying upon commonly available tools.

A noteworthy strength of the FISSEQ method, meanwhile, is that it promises to reveal the spatial substructure of the transcriptome. Knowledge of where particular transcripts reside in cells could also enable a guilt-by-association approach to identifying functional relationships between genes. With FISSEQ, one exciting prospect is the high-resolution determination of the characteristic path of a transcript-type in the cell throughout its program. Imagining FISSEQ analysis on thousands of cells, the portfolio of locations of a particular transcript type may be interpreted to reveal its cellular program. Such a brightly lit transcriptome could perhaps unlock some of the remaining mysteries of the cell.

## Abbreviations

aaUTPs: Aminoallyl dUTPs; bp: Base pair; dNTP: Deoxyribonucleotide triphosphate; dUTP: Deoxyuridine triphosphate; FISSEQ: Fluorescent *in situ* RNA sequencing; IVT: *In vitro* transcription; PCR: Polymerase chain reaction; RNA-seq: RNA sequencing; SOLiD: Sequencing by oligonucleotide ligation and detection; TIVA: Transcriptome *in vivo* analysis.

## Competing interests

The authors declare that they have no competing interests.
